# 

**Published:** 1962-01

**Authors:** 


MEDICAL JOURNAL OF THE SOUTH-WEST

*(Incorporating the Bristol Medico-Chirurgical Journal)*

Established 1883

VOL. 77 (i) January 1962 NO 283

### Contents

DISEASE OF MAJOR CEREBRAL VESSELS . . . *J. L. G. Thomson* 1

THE FASCIAL FLAP OPERATION FOR INGUINAL HERNIA *Edric Wilson* 7

OPEN CARDIAC SURGERY DURING PROFOUND HYPOTHERMIA *A. J. G. Dickens* 11

TRANQUILLIZERS . . . . . . *R. E. Hemphill* 18

MALIGNANT EPENDYMOMA MASQUERADING AS TUBERCULOUS MENINGITIS . . . . *Clinico–Pathological Conference* 28

OBITUARY . . . . . . . . . 35

NOTES AND NEWS 38

APPOINTMENTS . . . . . . . . 38

NEWS FROM THE SOCIETIES . . . . . . 39

*PUBLISHED QUARTERLY*

*ANNUAL SUBSCRIPTION* 16/- *POST FREE*

PUBLISHED UNDER THE AUSPICES OF THE
BRISTOL MEDICO-CHIRURGICAL SOCIETY

### *Editorial Committee*

Dr. N. J. Brown, Southmead Hospital, Bristol. *Joint Editor.*
Dr. A. B. Raper, Department of Pathology, Bristol Royal Infirmary. *Joint Editor.*
Dr. J. Macrae, Ham Green Hospital.
Dr. R. C. Wofinden, Central Clinic, Bristol.
Mr. A. E. S. Roberts, Medical Library, University of Bristol. *Secretary.*

### *Local Correspondents*

*Bath* . . Mr. A. D. Bateman, 3 The Circus, Bath.
*Exeter* . . Dr. L. N. Jackson, Mount Jocelyn, Crediton, Devon.
*Gloucester* . Dr. R. F. Jarrett, 9 College Green, Gloucester.
*Plymouth* . Dr. C. R. Croft, Lexden, Hartley Av., Plymouth.
*Taunton* . Dr. M. McAlley, 1 The Crescent, Taunton.
*Torquay* . Dr. A. Robinson Thomas, 20 Devon Square, Newton Abbot, Devon.
*Truro* . . Dr. F. D. M. Hocking, St. Andrews, Perranporth, Cornwall.
*Yeovil* . . Mr. J. A. Vere Nicoll, Knapp House, Yeovil, Somerset.

### NOTICE TO CONTRIBUTORS

Original articles are invited, provided they have not been published elsewhere. Notes of forthcoming events, meetings held, degrees conferred, staff appointments, and other local news are welcomed. The editorial committee reserves the right to make literary corrections.

Illustrations should always be supplied ready for reproduction. All re-touchings or other operations required will be charged to the author. Line drawings should be drawn in Indian ink. We regret that we must ask authors to pay for making blocks, if this is necessary.

J. W. ARROWSMITH LTD., WINTERSTOKE ROAD,
BRISTOL.

### *Notes and News*

***Obituary:*** J. W. E. Snawdon, Hugh Hadfield Carleton.

### *Appointments*

Dr. Shirley A. Lewis has been appointed assistant M.O., Hornsey and Tottenham.

### *SOUTH WESTERN REGIONAL HOSPITAL BOARD*
### *OCTOBER*, 1961

| *Name* | *Appointment* |
|---|---|
| | ***BRISTOL*** |
| MEHTA, S. H., M.D. (BARODA) | Medical Registrar, Southmead Hospital. |
| PAUL, T. N., M.B., B.S. (CALCUTTA) | Registrar in Diseases of the Chest, Ham Green Hospital and Bristol Chest Clinic. |
| | ***NORTH GLOUCESTERSHIRE*** |
| MULCAHY, F., M.B., B.Ch., B.A.O. (N. IRELAND), D.M.R.D., F.F.R. | Consultant Radiologist, Gloucestershire Royal Hospital. |
| SAXTON, H. M., M.B., B.S. (LOND.), M.R.C.P., D.M.R.D., F.F.R. | Consultant Radiologist, Cheltenham General Hospital. |
| HILL, A. B., M.D. (LOND.) | Consultant Pathologist, North Gloucestershire Clinical Area. |
| CRAUSE, J., M.B., B.S. (LOND.), D.P.M. | Senior Registrar in Psychiatry, Horton Road and Coney Hill Hospitals. |
| GOODEY, G. T., M.B., B.S. (LOND.), D.(OBST.)R.C.O.G. | Registrar in Obstetrics and Gynaecology, Gloucestershire Royal Hospital. |
| | ***BATH*** |
| DIXON, M. F., M.R.C.P., D.P.M. | Consultant Psychiatrist, Roundway Hospital, Devizes. |
| KIRKUP, J. R., F.R.C.S. (ENG.) | Senior Registrar in Orthopaedic and Traumatic Surgery, Bath Group of Hospitals. |
| VALVIS, MARY, M.B., B.Ch., B.A.O., L.A.H. (DUBLIN), D.P.M. (LOND.) | Senior Registrar in Psychiatry, Roundway Hospital, Devizes. |
| MCINNES, I. E., F.R.C.S. | Surgical Registrar, Bath Group of Hospitals. |
| TURNER, EUNICE V., B.A., M.B., B.Ch., B.A.O., D.C.H. | Registrar in Psychiatry, Roundway Hospital, Devizes. |
| VAN DEN BRUL, P. J., M.B., B.S. (LOND.), D.A. | Anaesthetic Registrar, Bath Group of Hospitals. |
| | ***SOUTH SOMERSET*** |
| KEMP, S. W., M.R.C.S., L.R.C.P., D.A., F.F.A.R.C.S. | Consultant Anaesthetist, Yeovil General Hospital. |
| ROBERTSON, SHEILA, J. B., M.B., Ch.B. (EDIN.), D.A. (ENG.) | Anaesthetic Registrar, Taunton and Somerset Hospital. |
| | ***DEVON AND EXETER*** |
| FORTUNE, D. W., M.B., Ch.B. (BRISTOL) | Senior Registrar in Pathology, Royal Devon and Exeter Hospital/Westminster Hospital, London (joint appointment). |
| HEYWOOD-WADDINGTON, M. B., B.A., M.B., B.CHIR. (CANTAB.), F.R.C.S. (ENG.) | Registrar in Orthopaedic and Traumatic Surgery, Torbay Hospital/Princess Elizabeth Orthopaedic Hospital. |
| PHILLIPS, J. B., M.B., B.S. (SYDNEY) | Surgical Registrar, Torbay/Newton Abbot Hospitals. |

| *Name* | *Appointment* |
| --- | --- |
| WOTHERSPOON, H. G., M.B.Ch.B. (BRISTOL), D.C.H., D.(OBST.)R.C.O.G. (LOND.) | Registrar in Obstetrics and Gynaecology, Royal Devon and Exeter Hospital/Southmead Hospital, Bristol (exchange appointment). |
| BURDON, J. F., M.B., B.S. (DURHAM), D.A. | G.P. Anaesthetist, Paignton Hospital (one session weekly). |
| KELLY, G. E. P., M.B., B.Ch., B.A.O. (DUBLIN). | G.P. Anaesthetist, Newton Abbot Hospital (temporary appointment—one weekly session), and Teignmouth Hospital (one weekly session). |

*PLYMOUTH*

| | |
| --- | --- |
| LAKE, R. H. N., M.B., B.S. (LOND.), D.A., F.F.A.R.C.S. | Consultant Anaesthetist, Plymouth Clinical Area. |
| ATKINS, P., F.R.C.S. (ENG.) | Surgical Registrar, South Devon and East Cornwall Hospital, Plymouth. |
| KARPOUZAS, J., M.D. (ATHENS) | Paediatric Registrar, South Devon and East Cornwall Hospital, Plymouth/United Bristol Hospitals (joint appointment). |
| LILAUWALA, N. F., D.C.H. (LOND.), M.R.C.P. (LOND. & EDIN.), F.R.C.P. (EDIN.) | Registrar in Psychiatry, Moorhaven Hospital, Ivybridge, S. Devon. |
| PALOSCHI, G. B., M.D. (PAVIA) | Orthopaedic Registrar, Mount Gold Hospital, Plymouth (one year only). |
| WARREN, J. D., M.D. (MANITOBA) | Orthopaedic Registrar, Mount Gold Hospital, Plymouth (one year only). |

